# Acquired Pancreatic Arteriovenous Malformation

**DOI:** 10.5334/jbr-btr.863

**Published:** 2015-09-15

**Authors:** A. Van Holsbeeck, I. Dalle, K. Geldof, L. Verhaeghe, K. Ramboer

**Affiliations:** 1 Department of Radiology, AZ Saint-Lucas Hospital, Brugge, Belgium; 2 Department of Pathology, AZ Saint-Lucas Hospital, Brugge, Belgium

**Keywords:** Arteriovenous malformation, pancreatic

## Abstract

Pancreatic arteriovenous malformation is a rare vascular anomaly which may cause abdominal pain, acute pancreatitis, gastrointestinal bleeding and portal hypertension. Pancreatic arteriovenous malformation is mostly congenital; however secondary pancreatic arteriovenous malformation due to pancreatitis has been suggested by some authors. We encountered a case which can confirm this presumption. Several imaging modalities are useful for the diagnosis of pancreatic arteriovenous malformation, especially dynamic contrast-enhanced studies. Angiography is the most important diagnostic tool because of the dynamic features of this vascular lesion. Treatment is advised and consists of surgical resection and/or transarterial embolization.

Pancreatitis may be complicated with a spectrum of arterial and venous abnormalities. Arterial complications most frequently involve formation of a pseudoaneurysm [1]. Less frequently, pancreatitis causes arterial thrombotic occlusion, which can lead to splenic or gastrointestinal infarction. Venous complications are relatively common and are related to splanchnic vein thrombosis. The splenic vein is especially vulnerable and approximately 50% of patients with splenic vein thrombosis will develop ‘left-sided’ portal hypertension with portoportal or portosystemic collaterals [2,3].

Pancreatic arteriovenous malformation (AVM) is a rare, mostly congenital, vascular anomaly. Several case reports have described pancreatic AVM as a cause for focal pancreatitis [4,5,6]. Conversely, some authors have suggested that pancreatitis may also be complicated with the formation of a pancreatic AVM [7,8].

We describe a case, the first to the best of our knowledge, which can clearly demonstrate a secondary pancreatic AVM due to pancreatic inflammation.

## Case report

**Figure 1:**
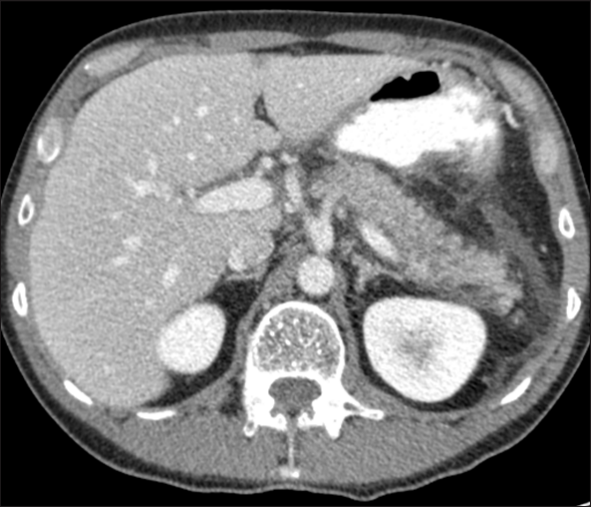
Contrast-enhanced axial CT scan (November 2006) showed edematous aspect of the pancreatic tail parenchyma with fluid in the anterior prerenal space, and in the anterior and posterior renal fascia.

A 48-year-old man with a history of alcohol abuse presented to the gastroenterology department for the first time in November 2006. He was suffering from severe epigastric pain and nausea for two days. Because the laboratory findings were suspicious for pancreatitis, the patient underwent computed tomography (CT) (Fig. 1). The examination showed an edematous pancreas tail and fluid in the anterior prerenal space and in the anterior, posterior and lateroconal fascia. There were no signs of chronic pancreatitis or vascular complications. In the following months the patient had two similar episodes of acute tail pancreatitis.

In September 2009 he presented with vague epigastric pain of several weeks duration. Serum amylase and lipase levels were normal, however the cholestatic parameters were mildly elevated. Therefore, a magnetic resonance (MR) study was performed. The examination demonstrated moderate chronic pancreatitis of the tail with atrophy and irregular bording of the Wirsung’s duct. There were no other abnormalities. Further examinations also revealed a Helicobacter pylori gastritis.

**Figure 2:**
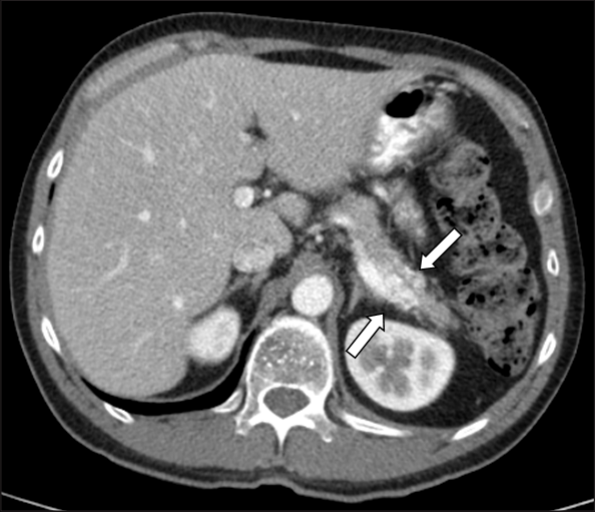
Contrast-enhanced axial CT scan (February 2012) showed a hypervascular lesion (white arrows) in the pancreatic tail in proximity to the splenic vein and measuring 2.5 cm × 2.4 cm × 1.7 cm.

**Figure 3:**
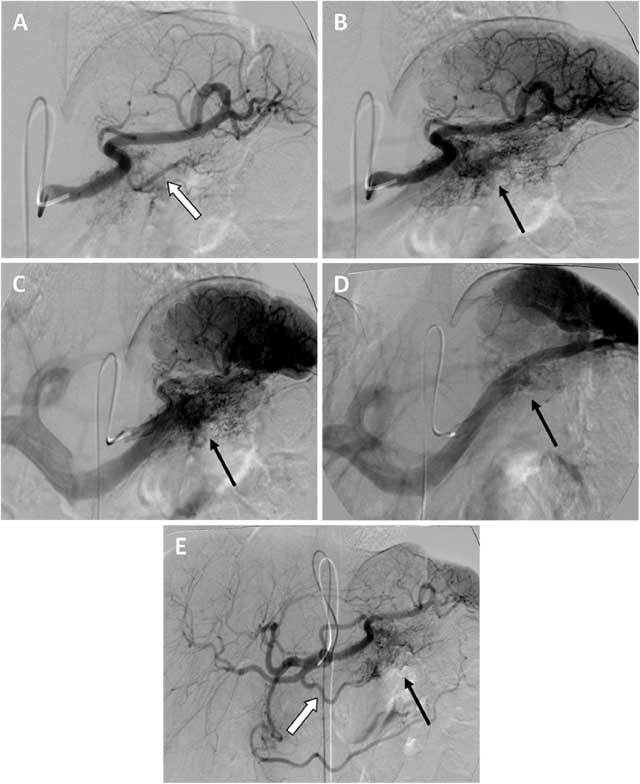
A Selective angiography of the splenic artery during arterial phase showed a prominent pancreatic branch of the splenic artery as feeding artery for the vascular lesion (white arrow). B Selective angiography of the splenic artery during late arterial phase demonstrated a racemose vascular network (black arrow). C Selective angiography of the splenic artery during early portal phase showed an early transient dense stain (black arrow). D Selective angiography of the splenic artery during portal phase showed early wash-out of the lesion (black arrow). E Angiography of the celiac trunk during late arterial phase revealed the dorsal pancreatic artery (white arrow) as a second important feeding vessel for the vascular lesion (black arrow). Note the anatomical variation with a proximal bifurcation of the common hepatic artery.

**Figure 4:**
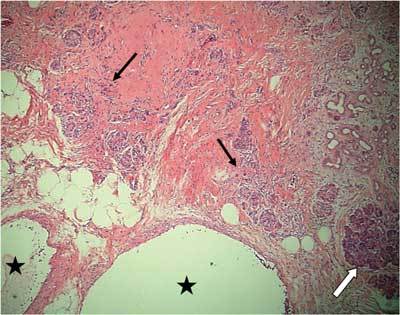
Histopathology of the resected specimen revealed numerous abnormally dilated blood vessels (asterisks). Several areas showed destruction of the exocrine pancreatic tissue with remaining islets of Langerhans and fibrotic changes of the parenchyma (black arrows). There was also some lymphocyte infiltration in these affected areas. Other areas showed relatively preserved exocrine pancreatic tissue (white arrow). The findings were consisting with pancreatic AVM and chronic pancreatitis. (Original magnification × 50).

In February 2012 the patient presented again to the gastroenterologist with epigastric and left upper quadrant pain. Laboratory findings were not suspect for acute pancreatitis. However, contrast-enhanced CT scan was performed in order to evaluate the known chronic pancreatitis and other causes of pain (Fig. 2). This study surprisingly revealed a hypervascular lesion in the pancreatic tail consisting of a conglomeration of small hypervascular spots and blood vessels. Further examinations were performed in order to differentiate a vascular malformation from a hypervascular pancreatic tumor, especially an islet cell tumor or a hypervascular metastasis. Contrast-enhanced MR study showed a focal area of heterogeneous contrast enhancement without an apparent nodular component. The subsequent angiography showed two large feeding arteries in the early arterial phase, followed by a racemose vascular network, an early transient dense parenchyma stain in the early portal phase and early wash-out of the lesion in the portal phase (Fig. 3). Based on these findings, a pancreatic arteriovenous malformation was suggested. The patient was treated with a resection of the pancreas tail and postoperative recovery was uneventful. Histopathologic examination revealed sequelae of chronic pancreatitis and numerous dilated blood vessels accompanied by blood clot formation and intimal hyperplasia (Fig. 4). The histopathological findings were consisting with a pancreatic AVM.

## Discussion

Pancreatic AVM is a vascular anomaly with an abnormal anastomosis of the arterial and portal network and was first described by Halpern et al in 1968 [9]. This vascular lesion is considered to be extremely rare with various clinical manifestations. Patients can be asymptomatic, but they may also be complicated with gastrointestinal bleeding, portal hypertension and abdominal pain [10]. Also several cases reported acute pancreatitis caused by a pancreatic AVM. The pancreatitis is thought to be caused by bleeding from the AVM into the pancreatic duct or by ischemia of the tissue due to vascular steal of the AVM [4,5,6].

Pancreatic AVM can be either primary, caused by abnormal development of the arteriovenous plexus in the embryo, or it can develop secondary. A review showed that pancreatic AVM is congenital in more than 90% of cases [11]. There is also an association with Rendu-Osler-Weber disease in 10%-30% of cases [12]. Only a few reported cases are considered secondary pancreatic AVM or pancreatic arteriovenous fistula, usually due to trauma, pancreas transplantation, tumor or inflammation [13,14,15]. It seems reasonable that the damage to pancreatic arterioles and venules, caused by the autodigestive process in pancreatitis, may cause small vascular connections with bypassing of the capillary bed. Some authors have suggested an acquired pancreatic AVM due to pancreatitis because histopathological examination revealed the AVM and findings of pancreatitis [7,8]. However, these authors cannot be conclusive because, as mentioned earlier, a congenital pancreatic AVM can also cause pancreatitis. Our case clearly shows that the pancreatic AVM was acquired due to pancreatitis, as previous imaging showed no vascular abnormalities.

Sonography of a pancreatic AVM typically shows multiple small hypoechoic nodules. Color Doppler sonography demonstrates a mosaic pattern of the lesion and pulsed Doppler sonography usually shows a pulsatile waveform in the portal vein instead of a continuous waveform. Doppler sonography is useful for differentiating a vascular anomaly from a cystic pancreatic tumor [5,16]. Ogawa et al. postulated that multiphase CT using a multislice CT scanner is helpful for the diagnosis of pancreatic AVM. The characteristic features include conglomeration of strong nodular stains and early enhancement of the portal venous system in the arterial phase. Further, multiphase contrast-enhanced CT can be helpful in identifying the feeding arteries of the vascular lesion, which is essential for treatment planning [8,17]. Contrast-enhanced MR study may show similar findings as a dynamic CT study. Further, T1- and T2-weighted images can demonstrate arteriovenous malformation as multiple signal voids [17]. Angiography is very useful for diagnosis because of the dynamic features of AVM. The findings include dilated feeding arteries in the arterial phase, followed by a complex intra-pancreatic vascular network resulting in a transient dense stain. Further there is early venous filling into the portal vein and early disappearance of the pancreatic stain, usually in the portal phase [4,8,18].

Differential diagnosis has to be made with hypervascular pancreatic tumors, such as islet cell tumor or hypervascular metastasis. Differentiation can be made with dynamic contrast studies because AVM shows early wash-out in the portal phase, whereas islet cell tumor and hypervascular metastasis are densely stained during the portal phase [8].

For the treatment of pancreatic AVM, surgical resection and transarterial embolization have been widely used. Gincul et al suggest transarterial embolization as a first therapeutic step [18]. However, several authors recommend early surgical resection because pancreatic AVM can cause portal hypertension, with the formation and possible rupture of portosystemic varices. Furthermore, once portal hypertension develops, it may be resistant to therapy, even if the AVM has been surgically removed. In addition, as pancreatic AVM may have multiple feeding arteries, embolization may be very difficult. Also recurrent gastrointestinal bleeding has been reported in symptomatic patients treated by embolization alone [10,19]. Some authors suggested the combination of transarterial embolization and surgery, because preoperative embolization may reduce surgical risk by decreasing the portal flow [20,21].

In conclusion, pancreatitis may be complicated with several vascular abnormalities. We described a case of acquired pancreatic AVM due to pancreatitis, a complication which was already suggested by some authors. Dynamic contrast imaging, especially angiography, is useful in the diagnosis. Treatment is advised and consists of surgical resection and/or transarterial embolization.

## Competing Interests

The authors declare that they have no competing interests.

## References

[1] Weits T, Breumelhof R (2002). Pseudo-aneurysm of the gastroduodenal artery complicating pancreatitis. JBR-BTR.

[2] Mendelson RM, Anderson J, Marshall M, Ramsay D (2005). Vascular complications of pancreatitis. ANZ J Surg.

[3] Mallick IH, Winslet MC (2004). Vascular complications of pancreatitis. JOP.

[4] Choi JK, Lee SH, Kwak MS (2010). A case of recurrent acute pancreatitis due to pancreatic arteriovenous malformation. Gut and Liver.

[5] Koito K, Namieno T, Nagakawa T (2001). Congenital arteriovenous malformation of the pancreas: its diagnostic features on images. Pancreas.

[6] Kanno A, Satoh K, Kimura K (2006). Acute pancreatitis due to pancreatic arteriovenous malformation: 2 cases reports and review of the literature. Pancreas.

[7] Uda O, Aoki T, Tsuchida A (1999). Pancreatic arteriovenous malformation observed to bleed from the bile duct and a duodenal ulcer: report of a case. Surg Today.

[8] Chang S, Lim HK, Lee WJ, Choi D, Jang K-T (2004). Arteriovenous malformation of the pancreas in a patient with gastrointestinal bleeding: helical CT findings. Abdom Imaging.

[9] Halpern M, Turner AF, Citron BP (1968). Hereditary hemorrhagic telangiectasia: an angiographic study of abdominal visceral angiodysplasias associated with gastrointestinal hemorrhage. Radiology.

[10] Song KB, Kim SC, Park JB (2012). Surgical outcomes of pancreatic arteriovenous malformation in a single center and review of literature. Pancreas.

[11] Nishiyama R, Kawanishi Y, Mitsuhashi H (2000). Management of pancreatic arteriovenous malformation. J Hepatobiliary Pancreat Surg.

[12] Butte JM, San Francisco IF, Pacheco F (2007). Arteriovenous malformation of the pancreas: report of a case. Surg Today.

[13] Ponsky JL, Hoffman M, Rhodes RS (1979). Arteriovenous fistula and portal hypertension secondary to islet-cell tumor of the pancreas. Surgery.

[14] Barth MM, Khwaja K, Faintuch S, Rabkin D (2008). Transarterial and transvenous embolotherapy of arteriovenous fistulas in the transplanted pancreas. J Vasc Interv Radiol.

[15] Trapp RG, Breuer RI, Crampton AR (1979). Pancreatic duct arteriovenous fistula and the metastatic fat necrosis syndrome. Dig Dis Sci.

[16] Yoon J-H, Han S-S, Cha S-S, Lee S-J (2005). Color Doppler ultrasonography of a pancreatic arteriovenous malformation. J Ultrasound Med.

[17] Ogawa H, Itoh S, Mori Y (2009). Arteriovenous malformation of the pancreas: assessment of clinical and multislice CT features. Abdom Imaging.

[18] Gincul R, Dumortier J, Ciocirlan M (2010). Treatment of arteriovenous malformation of the pancreas: a case report. Eur J Gastroenterol Hepatol.

[19] Aida K, Nakamura H, Kihara Y (2002). Duodenal ulcer and pancreatitis associated with pancreatic arteriovenous malformation. Eur J Gastroenterol Hepatol.

[20] Hosogi H, Ikai I, Hatano E (2006). Pancreatic arteriovenous malformation with portal hypertension. J Hepatobiliary Pancreat Surg.

[21] Rezende MB, Bramhall S, Hayes T (2003). Pancreatic arteriovenous malformation. Dig Surg.

